# Invasive Ductal Breast Carcinoma Metastasis to the Uterine Cervix Presenting As Heavy Vaginal Bleeding: A Case Report and a Literature Review

**DOI:** 10.7759/cureus.62523

**Published:** 2024-06-17

**Authors:** Gabriel Boudagh, Aria Kieft, Moumita S. R Choudhury, Suzanne M Jacques, Steven Miller

**Affiliations:** 1 Department of Radiation Oncology, Wayne State University School of Medicine, Detroit, USA; 2 Department of Pathology, Detroit Medical Center, Wayne State University, Detroit, USA

**Keywords:** diagnostic challenge, breast and gynaecological pathology, comprehensive physical exam, rare clinical presentation, metastatic breast disease

## Abstract

Breast carcinoma metastasis to the uterine cervix is a rare occurrence with diagnostic intricacies. We present a case of a 38-year-old woman diagnosed with bilateral stages IIIA and IIIB invasive ductal carcinoma of the breast who developed heavy vaginal bleeding post-treatment, revealing metastatic involvement of the cervix, confirmed by CT imaging and pathological examination, as the presenting sign of widely metastatic disease. This case underscores the importance of a thorough review of systems and physical exams as well as considering uncommon metastatic sites in breast cancer patients.

## Introduction

Breast cancer is the most common non-skin malignancy among women in the United States, constituting the second leading cause of cancer-related mortality in this demographic [1]. Early-stage breast cancer is curable, with a relative five-year survival rate of 99% for stage I, 93% for stage II, and 75% for stage III; unfortunately, metastatic breast cancer continues to have a poor prognosis at 29% [1]. Therefore, understanding the presenting signs and symptoms of breast cancer metastasis is of paramount importance, given its pivotal role in determining patient outcomes.

While variation in metastatic pattern exists between subtypes of breast cancer, bone, lung, brain, and liver comprise the most frequent sites of breast cancer metastasis [2]. Metastatic breast cancer affecting the uterine cervix is rare, with rates of metastasis to gynecologic organs from invasive lobular and invasive ductal carcinoma at 4.5% and 0.8%, respectively [3].

We present the case of a 38-year-old female patient diagnosed with metastatic invasive ductal carcinoma involving the cervix after completing neoadjuvant chemotherapy, radiotherapy, and bilateral mastectomy.

## Case presentation

The patient initially received a diagnosis of stage IIIA left breast cancer, with a clinical stage of T3 (tumor is more than 5 cm across), clinical stage N2a (clinically fixed or matted axillary lymph nodes), with weakly positive estrogen receptor and progesterone receptor (ER/PR) and human epidermal growth factor receptor 2 (HER2) positive left breast invasive ductal carcinoma in September 2022 [4]. She presented with an axillary mass noticed while nine weeks pregnant. Diagnostic work-up initially included a targeted ultrasound of the left breast with a biopsy. After the therapeutic termination of pregnancy, staging work-up included a CT of the chest, abdomen, and pelvis as well as a brain MRI and nuclear medicine (NM) bone scan, which were negative for metastatic disease. Commencing in early 2023, she underwent five cycles of neoadjuvant Taxotere (docetaxel), Paraplatin (carboplatin), Herceptin (trastuzumab), and Perjeta (pertuzumab) (TCHP). She was later transitioned to six cycles of adjuvant chemotherapy following disease progression between cycles 4 and 5. The response was assessed with bilateral diagnostic mammography, demonstrating no significant improvement in the left breast and Breast Imaging-Reporting and Data System 3 (BIRADS 3) tubular calcifications in the right breast. She was not a surgical candidate after chemotherapy, so the multidisciplinary tumor board recommended a course of neoadjuvant radiation therapy. In June 2023, a total of 5000 centigray (cGy) in 25 fractions with a 1000 cGy boost was delivered to the left breast and lymph regional lymph nodes alongside the resumption of Herceptin/Perjeta. Restaging CT of the chest, abdomen, and pelvis in July 2023 demonstrated interval improvement in the left breast cancer, interval development of extensive right breast malignancy, and no evidence of malignancy in the abdomen or pelvis. NM bone scan was completed on the same day and was read as negative for osseous metastases. Subsequently, the patient underwent a modified radical mastectomy of the left breast in August 2023. Final pathologic staging was reported as ypT1a and ypN1a in the left breast.

The second primary breast cancer of the right breast was found to be stage IIIB, clinical stage T4d (inflammatory carcinoma-breast was erythematous and edematous, marked skin thickening noted on mammography), stage N1 (multiple mobile lymph nodes palpated and assess on with ultrasound in the axilla), with weekly positive ER, negative PR, positive HER2 [4]. Repeat CT of the chest, abdomen, and pelvis demonstrated no evidence of metastatic disease. She underwent neoadjuvant radiation therapy to the right breast in October 2023 and a right mastectomy in December 2023. The final pathologic staging was ypT1a ypN2a. No additional imaging was completed between restaging in July and mastectomy in December.

Shortly after the right-sided mastectomy in December 2023, the patient reported mild abdominal pain and intermittent heavy vaginal bleeding, attributed to the irregularity of menses during chemotherapy. Upon presenting to the emergency department (ED) four days after the onset of symptoms, she was diagnosed with symptomatic anemia secondary to menorrhagia. A bedside ultrasound demonstrated minimal free abdominal fluid. She received a blood transfusion that alleviated her symptoms. She had a remote history of low-grade squamous intraepithelial lesion of the cervix in 2010 but no other abnormal pap smear history. She was admitted for further evaluation and was seen by a gynecologic oncologist. Unfortunately, she declined a pelvic examination at the time due to the absence of bleeding. She was discharged with outpatient follow with gynecology and primary care.

**Figure 1:**
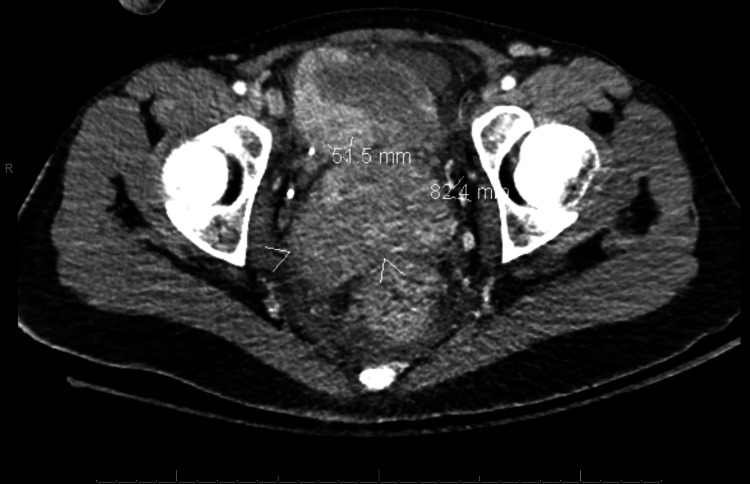
Axial CT scan displaying cervical mass White arrowheads bracket the cervical mass.

**Figure 2:**
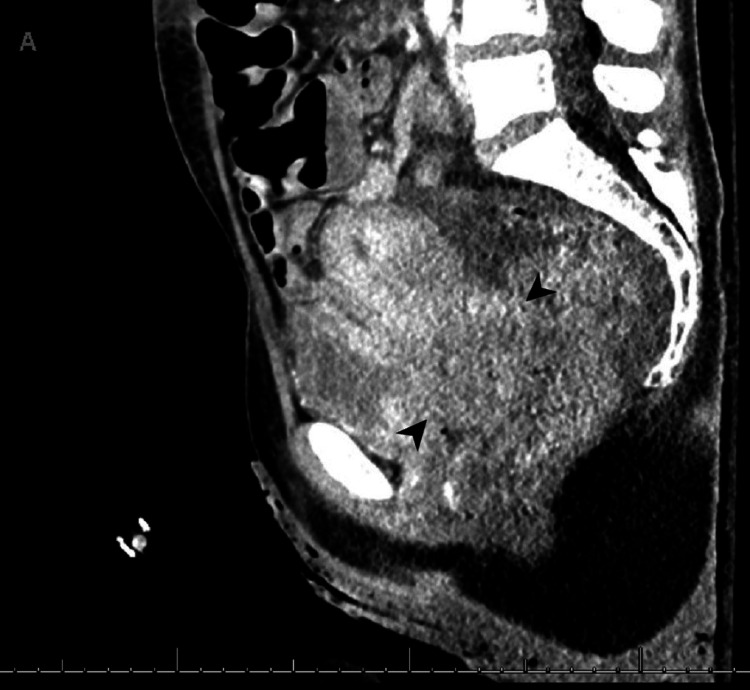
Sagittal view of cervical mass on contrast-enhanced CT scan Black arrowheads bracket the cervical mass.

Before the outpatient follow-up, the patient returned to the ED two weeks later with similar complaints. She was found to be hypotensive with a hemoglobin of 6.6 and received subsequent resuscitation with three units of packed red blood cells. A CT scan of the abdomen and pelvis with contrast (Figures 1-2) was performed after intravenous administration of 100 mL of Isovue-300, which revealed a sizable heterogeneous pelvic mass centered within the cervix, measuring approximately 8.2 x 5.2 cm alongside a 2.4 x 3.4 cm heterogeneously enhancing left adnexal mass.

The CT also revealed multiple peripherally enhancing nodular densities in the lower chest and left upper abdominal wall measuring up to 2.2 cm, which may represent soft tissue metastasis, as well as suspected lymphangitic carcinomatosis given right-sided presumed pleural metastatic deposits. Further imaging of the chest and abdomen later in the week would demonstrate evidence of widespread metastatic disease in the chest and liver. The patient was taken for an exam under anesthesia with a biopsy of the cervical mass.

**Figure 3:**
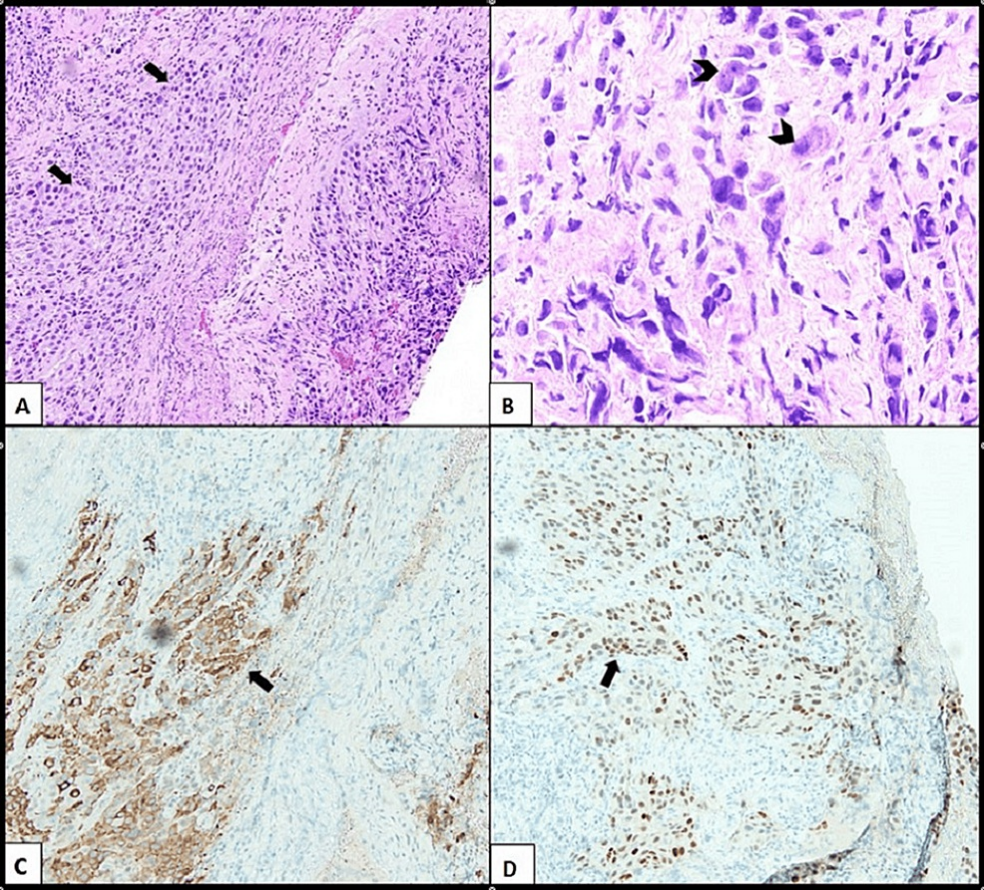
(A) The tissue is extensively involved by sheets of metastatic breast carcinoma with expansive growth pattern (arrows). There are no definite glandular or papillary features (H&E 200X). (B) The malignant cells exhibit nuclear enlargement and pleomorphism, often with prominent nucleoli (arrowheads) (H&E 400X). (C) Immunohistochemical staining for mammaglobin shows strong cytoplasmic staining (arrow) (100x). (D) Immunohistochemical staining for GATA-3 shows strong nuclear staining (arrow) (100X).

Grossly, the tissue from the cervical mass biopsy consisted of soft tissue fragments admixed with blood, in aggregate measuring 1.0 x 0.6 x 0.2 cm. Microscopically, the tissue showed extensive involvement by poorly differentiated carcinoma, characterized by solid expansile sheets of malignant cells with nuclear enlargement, pleomorphism, and a moderate amount of cytoplasm (Figures 3A-3B).

No definite glandular, squamous, or papillary features were seen. Immunohistochemical staining was strongly positive for CK7, a cytokeratin that supports a diagnosis of carcinoma, and for mammaglobin and GATA-3, markers that support a diagnosis of breast carcinoma (Figures 3C-3D).

Staining for BRST-2 was positive in rare cells. Staining for p40 and p63 was positive in only a few cells (nonspecific staining), and staining for CD10, PAX-8, and CK20 was negative, supporting the exclusion of other possible primary carcinomas, including squamous cell carcinoma and carcinoma of Müllerian origin. Immunohistochemical staining for p16 was also performed because it is frequently positive in primary cervical adenocarcinoma; however, it is also positive in many high-grade carcinomas, including high-grade breast carcinoma [5]. In this case, the p16 staining was positive in both the right and left primary breast carcinomas, as well as the cervical biopsy. The histologic features and immunohistochemical staining profile were consistent with metastatic breast carcinoma of no special type (WHO classification for a large group of invasive breast carcinomas that cannot be classified morphologically as any special type and that encompasses infiltrating duct carcinoma) [6]. Following this diagnosis, immunohistochemical studies for hormone receptors showed estrogen receptor 0.97% expression, progesterone receptor 1.68% expression, and HER2 score 3+ by immunohistochemistry (IHC), correlating with the known primary left breast ductal carcinoma.

**Figure 4:**
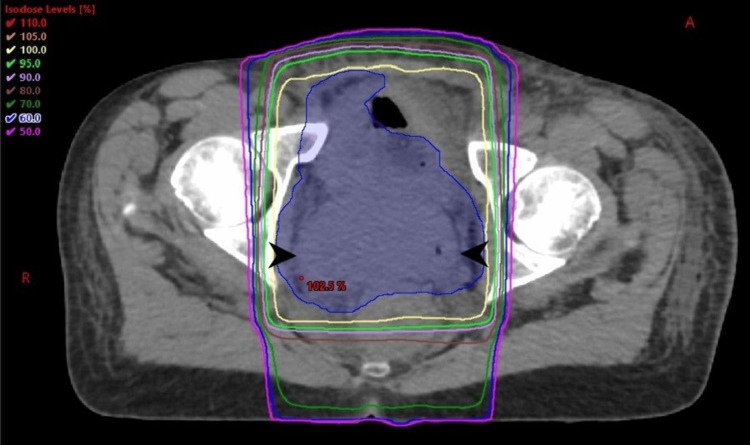
Axial CT planning scan of potential radiotherapy to control pain and bleeding in necrotic cervical mass Black arrowheads bracket cervical mass. The planning tumor volume (PTV) is indicated by blue-shaded volume. Radiation therapy was planned using a four-field 3D treatment technique. Isodose lines indicate the percentage of prescription radiation dose, as indicated by the legend at the top left.

The patient was referred to Radiation Oncology for further evaluation and treatment recommendations. Figure 4 shows an example of a potential treatment.

Unfortunately, before any additional treatment could be given, the patient passed away in February 2024 after developing pleural effusions and going into cardiac arrest.

## Discussion

Presented here is a noteworthy case exemplifying metastatic invasive ductal carcinoma afflicting the uterine cervix, an uncommon presentation. Despite metastases to the uterine cervix from non-gynecologic neoplasms being infrequent, comprising only 2% of gynecologic tract metastases, colorectal cancer typically leads in this context, followed by breast cancer. According to the National Cancer Institute, prevalent sites of breast cancer metastasis include the bone (30-60%), lung (21-32%), liver (15-32%), and brain (10%) [7]. Furthermore, the markedly higher proclivity of invasive lobular carcinoma compared to invasive ductal carcinoma to metastasize to gynecologic organs underscores the uncommon nature of the patient's manifestation [8]. Despite discernible differences between invasive lobular carcinoma and ductal carcinoma, such as the former's heightened tendency to be ER+/PR+ and its diffuse metastatic pattern versus the nodular pattern of the latter, the precise mechanistic underpinnings driving invasive lobular carcinoma's inclination towards gynecologic organs remain elusive [9].

Invasive breast carcinoma of no specific type is the most commonly diagnosed invasive breast carcinoma (75-80%), and arises from the epithelial cells at the terminal duct lobular unit [5]. The microscopic architecture in poorly differentiated tumors includes sheets, nests, or individual cells with a lack of tubular formations, as is seen in this case. Immunohistochemical studies aid in characterizing poorly differentiated carcinoma, particularly in our patient's case, as many metastatic deposits cannot be differentiated from new primary malignancy by H&E morphology alone. Mammaglobin, which is positive in this carcinoma, is expressed in 48-72.1% of breast carcinomas and directly correlates with a higher grade, and can help lead towards a diagnosis of a primary breast or gynecologic carcinoma [10]. GATA-3, also strongly positive in this carcinoma, is positive in 91-100% of hormone receptor-positive breast cancers, and 43-66% in triple-negative breast cancers [11].

Given the infrequency of invasive ductal carcinoma metastasis to the cervix, it was crucial to histologically confirm the nature of the patient’s mass via biopsy [8]. GATA-3, a pivotal transcription factor in breast epithelial differentiation, exhibits over 90% expression in primary and metastatic ductal and lobular carcinomas [12]. Positive staining of GATA-3 in the pathologic report supported the assertion of breast origin for the tumor. Moreover, positive staining of mammaglobin in the cervical tumor further corroborated its breast origin [9,13,14]. Negative staining of PAX8, a transcription factor linked with Müllerian system organogenesis, suggested no involvement of Müllerian-derived structures like the oviduct, uterus, cervix, or upper vagina [15]. Negative CK20 staining is used to assist in ruling out colorectal tumors, as well as mucinous ovarian carcinoma [16]. The diagnosis was made based on the constellation of all the immunohistochemical staining as well as clinical presentation. Staining for p16 was performed at a later date and found to be widely positive in both the primary breast tumor biopsies and the cervix.

As previously discussed, our comprehension of invasive ductal carcinoma metastasis to the cervix is primarily confined to case reports due to its rarity. Thus, it is imperative to scrutinize and discern trends and disparities among these cases. A concise literature review of reported cases of breast cancer metastases to the cervix, adapted from Horikawa et al. and Proença et al., revealed a total of 19 cases [17,18]. In this literature review (Table 1), of the 19 patients - including our case - there was an almost equal distribution of invasive lobular carcinoma and invasive ductal carcinoma, 11 and 8 cases, respectively [17-32].

**Table 1:** Review of findings in 18 cases in addition to our own IDC: invasive ductal carcinoma; ILC: invasive lobular carcinoma; AVB: abnormal vaginal bleeding; AAR: alive at the time of report; NM: no mention; DOD: died of disease

S. No.	Reference		Age	History	Presenting Symptoms	Survival
1	Song (1963) [19]		45	IDC	AVB	AAR (6 months)
2	Song (1963) [19]		49	IDC	AVB	AAR (4 months)
3	Lemoine and Hall (1986) [20]		39	IDC	AVB	6 months
4	Hepp et al. (1999) [21]		55	ILC	Abdominal pain	NM
5	Eichholz et al. (2004) [22]		52	ILC and IDC	AVB and pelvic pain	DOD
6	Salman et al. (2007) [23]		58	ILC	AVB	NM
7	Manci et al. (2008) [24]		41	ILC	none	AAR (20 months)
8	Bogliolo et al. (2010) [25]		78	ILC	none	AAR (2.5 years)
9	D'souza et al. (2010) [26]		44	ILC	AVB	NM
10	Horikawa et al. (2012) [17]		52	ILC	Abdominal discomfort	AAR (7 years)
11	Green et al. (2014) [27]		43	IDC	AVB	AAR (24 months)
12	Proença et al. (2016) [18]		58	ILC	none	DOD
13	Proença et al. (2016) [18]		77	IDC	none	DOD
14	Razia et al. (2017) [28]		58	ILC	AVB	AAR
15	Abdalla et al. (2018) [29]		32	IDC	AVB	NM
16	Cochrane et al. (2020) [30]		62	IDC	Dark vaginal discharge with AVB	NM
17	Qawasmeh et al. (2020) [31]		64	IDC	AVB	AAR (6 months)
18	Yuan et al. (2020) [32]		64	Adenocarcinoma	none	NM
19	Current study (2024)		38	IDC	AVB	DOD

This review demonstrated that presenting symptom leading to the discovery of metastasis to the cervix was abnormal vaginal bleeding in 12 out of 19 patients. Similar to the majority of symptomatic patients in the referenced table, our case also presented with abnormal vaginal bleeding nearly three weeks before the discovery of metastasis. However, during the first episode of abnormal vaginal bleeding, neoplasm was not high on the list of differential diagnoses, emphasizing the importance of vigilant monitoring for potential metastatic spread in patients with a history of malignancy. As the case noted, the patient had declined the initial pelvic exam in December after the workup was completed and the bleeding stopped. There is some evidence to suggest that breast cancer metastases to the uterine cervix may be underreported as in one series 41% of metastases were found only on autopsy [21]. If the clinician had higher concern for malignancy and this had been adequately expressed to the patient, she may have made a different decision, leading to an earlier diagnosis.

Through the presentation of this case study, our objective is to augment the scant existing literature, aiming to heighten awareness regarding the possibility of rare metastatic lesions impacting gynecologic structures in individuals exhibiting unusual symptoms. Moreover, we aspire to enrich the current corpus of knowledge concerning the assessment, management, and prognosis of metastatic invasive ductal carcinoma originating from the breast.

## Conclusions

In conclusion, this case report contributes to the existing literature on breast carcinoma metastasis to the cervix, shedding light on diagnostic challenges and emphasizing the importance of vigilant monitoring and comprehensive evaluation in breast cancer patients. This case urges healthcare professionals to maintain a high index of suspicion for potential malignancies in women with abnormal vaginal bleeding. A thorough evaluation, including a pelvic exam, is crucial for early diagnosis and improved patient outcomes. Additionally, given that the patient had declined the pelvic exam in December before her resuscitation attempt, communication and education are also essential to empower patients to make informed decisions. Further research and awareness are warranted to understand better the mechanisms and implications of metastatic spread to uncommon sites, ultimately improving patient care and outcomes.

## References

[1] Giaquinto AN, Sung H, Miller KD (2022). Breast cancer statistics, 2022. CA Cancer J Clin.

[2] Arciero CA, Guo Y, Jiang R, Behera M, O'Regan R, Peng L, Li X (2019). ER+/HER2+ breast cancer has different metastatic patterns and better survival than ER-/HER2+ breast cancer. Clin Breast Cancer.

[3] Borst MJ, Ingold JA (1993). Metastatic patterns of invasive lobular versus invasive ductal carcinoma of the breast. Surgery.

[4] Giuliano AE, Edge SB, Hortobagyi GN (2018). Eighth edition of the AJCC Cancer Staging Manual: breast cancer. Ann Surg Oncol.

[5] Shin E, Jung WH, Koo JS (2015). Expression of p16 and pRB in invasive breast cancer. Int J Clin Exp Pathol.

[6] WHO Classification of Tumors Editorial Board (2019). Breast Tumors.

[7] Lokadasan R, Ratheesan K, Sukumaran R, Nair SP (2015). Metastatic lobular carcinoma of breast mimics primary cervix carcinoma: two case reports and a review of the literature. Ecancermedicalscience.

[8] Waks AG, Lennon J, Yadav BS (2015). Metastasis to the cervix uteri 15 years after treatment of lobular carcinoma of the breast. Semin Oncol.

[9] Mazur MT, Hsueh S, Gersell DJ (1984). Metastases to the female genital tract: analysis of 325 cases. Cancer.

[10] Wang Z, Spaulding B, Sienko A (2008). Mammaglobin, a valuable diagnostic marker for metastatic breast carcinoma. Int J Clin Exp Pathol.

[11] Krings G, Nystrom M, Mehdi I, Vohra P, Chen YY (2014). Diagnostic utility and sensitivities of GATA3 antibodies in triple-negative breast cancer. Hum Pathol.

[12] Cimino-Mathews A, Subhawong AP, Illei PB (2013). GATA3 expression in breast carcinoma: utility in triple-negative, sarcomatoid, and metastatic carcinomas. Hum Pathol.

[13] Lu S, Yakirevich E, Wang LJ, Resnick MB, Wang Y (2019). Cytokeratin 7-negative and GATA binding protein 3-negative breast cancers: clinicopathological features and prognostic significance. BMC Cancer.

[14] Fiel MI, Cernaianu G, Burstein DE, Batheja N (1996). Value of GCDFP-15 (BRST-2) as a specific immunocytochemical marker for breast carcinoma in cytologic specimens. Acta Cytol.

[15] Xiang L, Kong B (2013). PAX8 is a novel marker for differentiating between various types of tumor, particularly ovarian epithelial carcinomas. Oncol Lett.

[16] Prat J (2005). Ovarian carcinomas, including secondary tumors: diagnostically challenging areas. Mod Pathol.

[17] Horikawa M, Mori Y, Nagai S, Tanaka S, Saito S, Okamoto T (2012). Metastatic breast cancer to the uterine cervix mimicking a giant cervical leiomyoma. Nagoya J Med Sci.

[18] Proença S, Reis MI, Cominho J, Conde PC, Santos E Pereira H, Ribeiro FC (2016). Metastatic breast cancer in uterine cervix: a rare presentation. J Low Genit Tract Dis.

[19] Song J (1963). Metastatic carcinoma of the uterine cervix from primary breast cancer. JAMA.

[20] Lemoine NR, Hall PA (1986). Epithelial tumors metastatic to the uterine cervix. A study of 33 cases and review of the literature. Cancer.

[21] Hepp HH, Hoos A, Leppien G, Wallwiener D (1999). Breast cancer metastatic to the uterine cervix: analysis of a rare event. Cancer Invest.

[22] Eichholz ACK, Geisler JP, Sood AK (2003). Metastatic breast cancer to the uterus and cervix. J Gynecol Surg.

[23] Salman T, Massiah N, Burns S, Mills S (2007). Metastatic breast cancer to the cervix and myometrium. J Obstet Gynaecol.

[24] Manci N, Marchetti C, Esposito F (2008). Late breast cancer recurrence to the uterine cervix with a review of the literature. Int J Gynecol Pathol.

[25] Bogliolo S, Morotti M, Valenzano Menada M, Fulcheri E, Musizzano Y, Casabona F (2010). Breast cancer with synchronous massive metastasis in the uterine cervix: a case report and review of the literature. Arch Gynecol Obstet.

[26] D'souza MM, Sharma R, Tripathi M, Saw SK, Anand A, Singh D, Mondal A (2010). Cervical and uterine metastasis from carcinoma of breast diagnosed by PET/CT: an unusual presentation. Clin Nucl Med.

[27] Green AE, Biscotti C, Michener C, Belinson J (2004). Isolated cervical metastasis of breast cancer: a case report and review of the literature. Gynecol Oncol.

[28] Razia S, Nakayama K, Tsukao M (2017). Metastasis of breast cancer to an endometrial polyp, the cervix and a leiomyoma: a case report and review of the literature. Oncol Lett.

[29] Abdalla AS, Lazarevska A, Omer MM, Tan E, Asaad A, Sathananthan S (2018). Metastatic breast cancer to the cervix presenting with abnormal vaginal bleeding during chemotherapy: a case report and literature review. Chirurgia (Bucur).

[30] Cochrane E, Kim S, Kudelka A, Burke W (2020). Invasive ductal breast carcinoma metastasis to the cervix: a case review and clinical correlation. Gynecol Oncol Rep.

[31] Qawasmeh J, A-Hussaini M, Koro S (2020). Invasive ductal carcinoma of the breast with metastasis to the uterine cervix. Clin Case Rep Int.

[32] Yuan L, Oshilaja O, Sierk A, Zhang G, Booth CN, Brainard J, Dyhdalo KS (2021). Metastatic breast cancer diagnosed on cervical cytology. Cytopathology.

